# *Aspergillus* in the Indoor Air of Critical Areas of a Tertiary Hospital in Brazil

**DOI:** 10.3390/jof10080538

**Published:** 2024-08-01

**Authors:** Michele Scardine Corrêa de Lemos, Minoru German Higa Junior, Anamaria Mello Miranda Paniago, Marcia de Souza Carvalho Melhem, Juliana Possato Fernandes Takahashi, Wellington Santos Fava, Fabio Antonio Venancio, Nayara Moreno Martins, Marilene Rodrigues Chang

**Affiliations:** 1 Graduate Program in Infectious and Parasitic Diseases, Faculty of Medicine, Federal University of Mato Grosso do Sul, Campo Grande 79070-900, MS, Brazil; michele.scardine@eb.mil.br (M.S.C.d.L.); anamaria.paniago@ufms.br (A.M.M.P.); marcia.melhem@ufms.br (M.d.S.C.M.); wellington.fava@ufms.br (W.S.F.); fabio.venancio@uems.br (F.A.V.); 2 Hospital Infection Control Commission, Maria Aparecida Pedrossian University Hospital, Federal University of Mato Grosso do Sul, Campo Grande 79070-900, MS, Brazil; minoru.junior@ebserh.gov.br; 3 Quantitative Pathology Unit, Adolfo Lutz Institute, Secretary of Health, São Paulo 01246-002, SP, Brazil; juliana.takahashi@ial.sp.gov.br; 4 Laboratory of Infectious and Parasitic Diseases, Faculty of Medicine, Federal University of Mato Grosso do Sul, Campo Grande 79070-900, MS, Brazil; 5 Microbiological Research Laboratory, Faculty of Pharmaceutical Sciences, Food and Nutrition, Federal University of Mato Grosso do Sul, Campo Grande 79070-900, MS, Brazil; nayara.moreno@ufms.br

**Keywords:** Aspergillosis: airborne fungi, *Fumigati*, *Nidulantes*, *Nigri*, *Flavi*, *Terrei*, air contamination intensive care unit

## Abstract

Airborne *Aspergillus* spp. are critical pathogens that cause nosocomial infections in hospitals. Despite their importance, little is known about the distribution of *Aspergillus* species in the indoor air of hospitals in Brazil. We investigated *Aspergillus* spp. in the indoor air of critical areas in a tertiary hospital in Brazil. Air samples (n = 238) were collected from the intensive care unit (ICU), medical clinic unit (MCU), and urgency and emergency unit (UEU) using an air sampler (100 L/min). Of the 324 *Aspergillus* isolates, 322 were identified using phenotypic methods, and 37 were identified using DNA sequencing. *Aspergillus* spp. was grouped into five sections: *Fumigati* (29.3%), *Nidulantes* (27.8%), *Nigri* (27.5%), *Flavi* (11.7%), and *Terrei* (3.1%). The predominant species identified via sequencing were *Aspergillus sydowii* (n = 9), *Aspergillus flavus* (n = 7), and *Aspergilus fumigatus* (n = 6). The number of *Aspergillus* spp. and their sections varied according to the collection day. *A. fumigatus* was isolated more frequently during winter and in the ICU. This study is the first to demonstrate the diversity of airborne *Aspergillus* (saprophytic, allergenic, toxigenic, and potentially pathogenic) strains in a hospital located in the Midwest region of Brazil. It contributes to the knowledge of the diversity of cryptic species in the hospital environment.

## 1. Introduction

An environment contaminated with fungal spores plays an important role in the colonization of patients, and can precede infections such as invasive aspergillosis, a potentially lethal condition if not diagnosed and treated promptly [1,2,3,4].

*Aspergillus* spp. is responsible for the second highest occurrence of invasive fungal infections in tertiary hospitals [5]. These opportunistic fungi can cause allergic reactions and serious pathologies, such as pneumonia, aspergilloma, and even disseminated infections, depending on the individual’s immunological condition [2,6,7,8]. The increases in incidence, resistance to antifungals, and mortality due to invasive aspergillosis are worrying trends [3,9,10,11,12].

*Aspergillus* species belonging to sections *Nigri*, *Fumigati*, and *Flavi* are most commonly described in hospital environments and clinical samples [2,3,5,7,13,14,15]. *A. fumigatus* is more prevalent than the other species, and an increase in antifungal-resistant isolates from clinical and environmental samples has been reported [3,7,11,12]. Despite its significance, little is known about the distribution of airborne *Aspergillus* species in Brazilian hospitals [1]. *Aspergillus niger, A. fumigatus,* and *A. flavus* were the most commonly found [14].

Approximately 62% *of Aspergillus* isolates in published Brazilian studies have not been identified at the species level [14]. This study describes for the first time the different sections and species of *Aspergillus* in the indoor air of critical hospital areas in the state of Mato Grosso do Sul, Brazil, contributing to our knowledge of the biodiversity of these microorganisms in hospital environments.

## 2. Materials and Methods

### 2.1. Collection Procedure

Air samples were collected every 15 days in January, February, March, August, and December 2021 between 2:00 pm and 4:00 pm from Campo Grande, the capital of Mato Grosso do Sul State, in the Midwest region of Brazil. University Hospital Maria Aparecida Pedrossian (UHMAP) is a public tertiary teaching hospital. Geographically, Campo Grande is located near the borders of Brazil, Paraguay, and Bolivia. The tropical climate is characterized by dry winters and humid summers, with heavy rainfall and high temperatures. Humidity, temperature, and rainfall information were obtained from Mato Grosso do Sul Weather and Climate Monitoring Center website (https://www.cemtec.ms.gov.br/bancodedados/2021-2), accessed on 21 July 2022 and corresponded to the days and times of collection.

Air collection was performed in the intensive care unit (ICU), in the medical clinic unit (MCU), and in the urgency and emergency unit (UEU). The MCU and the UEU do not have air conditioning. The ICU is equipped with wall air conditioners, which are periodically cleaned and maintained by a specialized company. None of the three units has a high efficiency air filtration system. Floors and surfaces are cleaned daily.

Of each hospitalization unit, nine air samples/day were collected using an air impactor (MiniCapt Microbial Air Sampler, Particle Measuring Systems, Boulder, CO, USA) adjusted to a volume of 100 L/min per 1 min on a plate containing Dichloran Rose-Bengal Chloramphenicol (DRBC) agar (Kasvi, Conda Laboratories, Madrid, Spain). Immediately after collection, petri dishes containing air samples were packed in individual plastic bags and transported to the laboratory in a closed box. The fungal colony plates were processed in a biological safety cabin to avoid possible sample contamination. The plates were then incubated at 30 °C for 72 h. In this study, fungal load corresponds to the number of filamentous fungi colony-forming units (CFU/m^3^) of a Petri dish.

### 2.2. Fungal Identification

The collection sites were positioned 1.5 m above the ground and air was collected at the same points determined during the first sampling.

*Aspergillus* isolates (n = 324) were identified at the section level by analyzing their macroscopic and microscopic characteristics [16]. Owing to financial constraints, only 37 isolates were randomly selected for molecular identification using polymerase chain reaction (PCR) and sequencing. Briefly, genomic DNA was extracted using a Biogene kit (Bioclin, Belo Horizonte, Brazil), following the manufacturer’s instructions. PCR was performed as previously described [17,18]. The isolated DNA fragments were amplified using primers targeting the *β-tubulin* and *calmodulin* regions (Table 1). The PCR products were purified using the ExoSap-IT Express kit (Applied Biosystes, Foster City, CA, USA) and sequenced using *Bt*2a/*Bt*2b primers in an ABI 3730xl System service provided by Macrogen facility (Seoul, South Korea). The sequences were checked for quality, and the forward and reverse sequences were concatenated using Geneious software (Geneious 7.1.3, Biomatters Ltd., Auckland, New Zealand). For species identification, nucleotide sequence comparisons were performed against the National Center for Biotechnology Information (NCBI) database using the Basic Local Alignment Search Tool (BLAST; GenBank accession OR758882-OR758901.

### 2.3. Statistical Analyses

ANOVA was used to evaluate whether there was a significant difference in the number of *Aspergillus* spp. collected per plate across collection days. The Pearson chi-square or the Fisher exact test (in cases where one of the cells had an expected value of ≤5) was applied to determine whether there was a difference in the type of *Aspergillus* collected from different hospital units and between seasons.

A Pearson correlation analysis was conducted to assess the influence of independent variables (humidity, temperature, and rainfall) on the dependent variable, total *Aspergillus* spp. The dataset comprises observations collected on different dates during the study period. Correlation coefficients (r) were used to measure the strength and direction of the relationships between independent and dependent variables. The *p* values were examined to determine the statistical significance of each correlation. Statistical analyses were performed using R (version 4.1.1) at the RStudio interface [21].

## 3. Results

Between January and December 2021, in the 238 air samples collected from the intensive care unit (ICU), Medical Clinic Unit (MCU), and the urgency and emergency unit (UEU), we observed the growth of 4300 filamentous fungi colonies.

### 3.1. Number of Colony-Forming Units and Identification of Aspergillus Sections and Species

#### 3.1.1. Number of Filamentous Fungi Colony-Forming Units (CFU)

A total of 4295 filamentous fungi CFU were isolated from indoor air of UHMAP critical areas. The average number of filamentous fungal colonies isolated was 224 CFU/m^3^ and the average number per air sample ranged from 7.4 to 42.4 CFU, with variations depending on the day of collection. Table 2 shows the filamentous fungal load in hospital air according to the collection date. A significant difference was found between the fungal load and the day of collection (*p* < 0.001).

Of the total colonies obtained, 324 (7.5%) exhibited macro- and microscopic characteristics of *Aspergillus* spp. and were grouped into five sections: *Fumigati* (n = 95, 29.3%), *Nidulantes* (n = 90, 27.8%), *Nigri* (n = 89, 27.5%), *Flavi* (n = 38, 11.7%), and *Terrei* (n = 10, 3.1%). Two isolates (0.6%) could not be identified in this study.

The overall average number of *Aspergillus* spp. isolated per air sample ranged from 17 to 30 CFU/m^3^, depending on the day of collection. The CFU of *Aspergillus* colonies varied between the days of collection (*p* = 0.02). The daily distribution of *Aspergillus* sections is presented in Table 3.

#### 3.1.2. *Aspergillus* Section Per Hospitalization Unit

**Figure 1:**
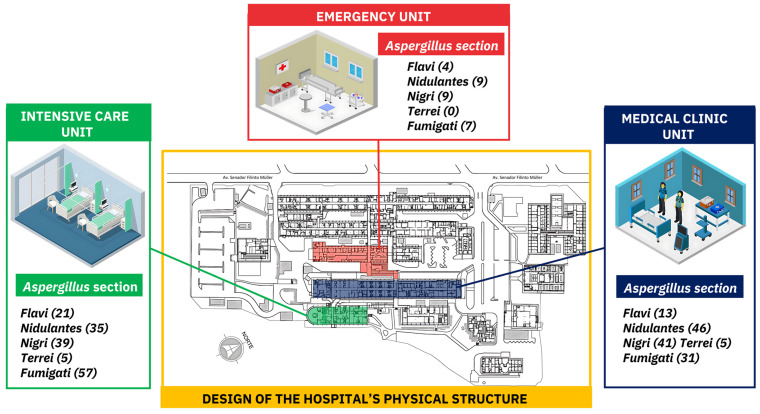
*Aspergillus* distribution according to section and collection site. UHMAP Campo Grande-MS, January–December 2021.

Analysis of the number of *Aspergillus* colonies per hospitalization unit revealed that the ICU had the highest load (157; 48.5%) of *Aspergillus* colonies dispersed in the air. *Fumigati* was the most frequent section of the ICU (36.3%, 57/157). *Aspergillus* from the *Nidulantes* section (46/138) was more prevalent in the MCU, followed by that in the *Nigri* section (41/138). Figure 1 shows the distribution of *Aspergillus* spp. sections per hospital unit.

#### 3.1.3. *Aspergillus* Load According to Section and Season

The variation in *Aspergillus* load according to section and season was significant (*p* = 0.001). Unfortunately, it was not possible to collect data during the autumn. Table 4 shows that *Aspergillus* species from the *Fumigati* section were more frequent in the winter (64, 67.4%). In contrast, species from the *Nigri* section (71; 79.8%) and *Nidulantes* (49; 54.4%) were isolated during the summer.

**Figure 2:**
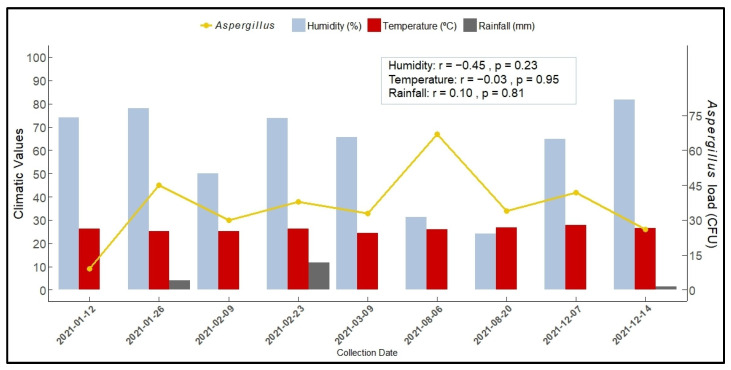
*Aspergillus* spp. load according to humidity, temperature, and rainfall on the date of collection. Campo Grande-MS, 2021. Note: Humidity (%) represents the percentage of humidity recorded on each collection date. Temperature (°C): Indicates the temperature in degrees Celsius observed on each collection date. Rainfall (mm): The amount of precipitation in millimeters measured on each collection date. *Aspergillus*: Presents the load of *Aspergillus* spp. on each collection date. Explanatory Note: This grouped bar chart illustrates the climatic variations and load of *Aspergillus* on nine different collection dates throughout 2021. Each group of bars represents measurements of humidity, temperature, and precipitation (rainfall) associated with a specific date. The above legend describes the climatic variables represented by distinct colors, and the gold color indicates the load of *Aspergillus*. The heights of the bars represent the values of each variable, whereas the secondary axis on the right displays the load of *Aspergillus*. Source: Mato Grosso do Sul State Weather and Climate Monitoring Center (CEMTEC/SEMAGRO). The Pearson correlation coefficient was used for correlation analysis.

Figure 2 shows the *Aspergillus* spp load according to humidity, temperature, and rainfall on the collection dates. In winter, during periods of lower rainfall, we observed a larger load of *Aspergillus* in ambient air. Pearson correlation analysis indicated that humidity, temperature, and rainfall were not significantly correlated with the presence of *Aspergillus* spp. in indoor air.

## 4. Discussion

The results showed saprophytic, allergenic, toxigenic, and potentially pathogenic *Aspergillus* strains in the air of critical areas of the studied hospital. This study provides for the first time the molecular identification of *Aspergillus* species from the indoor air of critical hospital areas in the Midwest region of Brazil. Globally, few studies have evaluated the microbial quality of indoor air in hospitals (or hospital environments).

Despite their relevance, there are no specific regulations or guidelines that specify limits for fungi in the indoor air of hospitals [22]. According to the standards of the Brazilian Ministry of Health and the National Health Surveillance Agency, the Maximum Recommended Value for fungal contamination of indoor air is 750 CFU/m^3^ in indoor air [23]. Although the average number of filamentous fungal colonies found in environmental air in our study was within the limits established by Brazilian law, this value (224 CFU/m^3^) was higher than those previously described in other Brazilian hospitals. Pantoja et al. (2012) [24] described that, in air collected from the ICUs of three hospitals in northeastern Brazil, the number of fungal spores ranged from 43.75 to 73.67 CFU/m^3^. In the indoor air of three ICUs of two university hospitals in southern Brazil, the median overall fungal concentrations for ICU 1, 2, and 3 were 109.5, 134.3, and 55.7 CFUm^3^, respectively [1].

In this study, the source of air contamination by *Aspergillus* spores is uncertain. External and internal environmental factors and other predisposing conditions may be involved. In the medical clinic unit (MCU), and in the emergency unit (UEU) where there is no air conditioning and the windows are open, there is the possibility that the indoor *Aspergillus* can, in part, originate from an outdoor environment via the airflow. In these hospital units, the movement of people can also favor the dispersion of fungal conidia that grow on surfaces [25]. In the ICU, where the movement of people is quite restricted, and there is no direct access to the external environment, it is believed that the aerocontamination by *Aspergillus* is mainly due to internal sources. In enclosed environments, without a high efficiency air filtration system or with an improperly maintained air system, bacteria and fungi can spread and survive in the environment [26,27].

Our results are particularly relevant for hospital units to which immunosuppressed patients and those with chronic diseases are admitted, such as intensive care units and medical clinics. The results revealed that the average number of filamentous fungal colonies (including *Aspergillus* spp.) per plate varied significantly, depending on the day of collection (*p* < 0.05). Previous studies have shown that in hospitals, where many spores can circulate in the air, the risks of both infection and hospital infection outbreaks increase significantly [1,26,28].

The findings of this study prompted the Hospital Infection Control Commission to act, as fungi are recognized as indicators of indoor air quality. Support from senior hospital management was sought to implement routine air analysis. Additionally, meetings were held with the engineering team to discuss interventions for enhanced environmental control, such as installing barriers to prevent air infiltration from construction or renovation activities, and regular maintenance of air conditioning equipment to reduce the amount of filamentous fungal spores.

Other measures to minimize the number of filamentous fungal spores in the internal air, include vacuum cleaning, restricting the entry and propagation of microorganisms in the internal environments, and using air cleaning devices. To control air quality more effectively and reduce fungal colony counts, it may be necessary to install heating, ventilation, and air conditioning (HVAC) and high efficiency particulate air (HEPA) filtering systems [13,22].

One of the limitations of this study is that for logistical reasons, outdoor air samples could not be obtained, which could have provided more accurate data on the distribution of these fungi in the environment. Sample collections were consistently conducted simultaneously prior to routine cleaning of the site to minimize potential sources of interference. The higher number of colonies observed on specific days may have been influenced by various internal or external factors, such as the movement of patients, healthcare teams, visitors, and employees, as well as minor renovations within the hospital [13,29,30].

As observed in the present study, filamentous fungi present in the internal air of hospitals may aggravate asthma and allergic rhinitis and cause serious diseases in susceptible individuals [2,4,9,31,32,33,34].

The analysis of the microbial load of fungi in indoor air is challenging. In this study, on the last day of collection (14 December 2021), the number of filamentous fungi was higher in almost all sectors, indicating that the air in the hospital had a high load of filamentous fungi. Future studies should aim to correlate the genotypes of clinical and environmental isolates collected during the same period to determine the impact of high concentrations of airborne spores dispersed in the air.

*Aspergillus* from the *Fumigati* section is considered the main cause of invasive aspergillosis and was the most prevalent agent in this study. Unlike what has been described in other countries and other Brazilian regions, in the studied hospital, *Aspergillus* from the *Nidulantes* section was the second most frequent, with a similar quantity to species from the *Nigri* section, which tends to be the second most frequent section in the indoor air of hospitals [12,13,15]. *Aspergillus* belonging to the *Fumigati* and *Nigri* sections was prevalent in the indoor air of UHMAP, corroborating studies in Asia [13], Europe [12,15], Africa [25], and the American continent [1]. In Brazil, these fungi have been described in hospital environments in the Northeast [24,27,35,36], Midwest [37], Southeast [38], and South [1,39] regions. Most Brazilian studies have not identified *Aspergillus* at the species level, limiting our knowledge of the most prevalent species in hospitals in Brazil [14,26].

In the present study, significant differences were observed in the load of sections of *Aspergillus* spp., according to the sampling date. In the first quarter of 2021, isolates from the *Nigri* and *Nidulantes* sections were predominant. This period was characterized by heavy rainfall. High humidity may have contributed to the many *Aspergillus* isolates found in these sections [8]. *Aspergillus* species from the *Nigri* and *Nidulantes* sections are routinely recovered from hospital environments and can cause severe diseases [8,31,40]

*Aspergillus* from the *Fumigati* section was the most common species isolated from ICU patients (36.3%). This finding is crucial because immunocompromised patients are vulnerable to fatal respiratory infections caused by *A. fumigatus* [4,9,41]. Species from the *Flavi* section were also frequently found in the ICU. According to a recent study, *A. flavus* is the second most common *Aspergillus* species isolated from invasive aspergillosis patients [10]. The presence of *Aspergillus* isolates from the *Fumigati* and *Flavi* sections in the critical care units found in our study highlights the urgent need to implement measures to improve air quality, not only in the units studied but also in other hospital units.

Previous studies have shown that temperature and relative humidity positively correlate with the growth and reproduction of fungi in hospital environments [42,43,44]. On December 14, considered summer in Brazil, the largest load of filamentous fungi found may be due to high relative humidity, which on this day was greater than 80%. However, in our study, the environmental parameters evaluated (humidity, temperature, and rainfall) did not significantly influence the load of *Aspergillus* spp. in the internal air of the hospital. Similar to our results, Cho et al., 2018 [13] did not find a statistical correlation between *Aspergillus* sections in the internal air and temperature. This result may be related to the remarkable ability of some species, such as *A. fumigatus*, to adapt to the changing climate [45]. In fact, little is known about the variations between *Aspergillus* strains and their growth at different temperatures and how their geographical origin affects such variations [45]. Further studies are needed to verify how these environmental parameters correlate with the presence of different *Aspergillus* species.

The study hospital is located in a tropical climate characterized by dry winters and humid summers with heavy rain and high temperatures. Although we did not observe a correlation between the number of *Aspergillus* isolates in the air and the temperature, it was observed that during the winter, when there was less rain, more CFU of *Aspergillus* spp. were isolated on average per plate. One of the limitations of this study was the inability to collect air samples during the autumn owing to the COVID-19 pandemic. The impossibility of collecting samples in autumn influenced the total number of filamentous fungi, the number of *Aspergillus* (sections and species) isolated, and other results related to seasonality. However, we could not evaluate the impact of the lack of such data in the present study. The reason some *Aspergillus* species are more prevalent in summer and winter is not well established. Seasonal fluctuations in airborne fungal concentrations are influenced by various factors, with geoclimatic conditions being a significant contributor [13,42,45].

Statistical analyses revealed differences in *Aspergillus* sections and sampling periods in terms of fungal cell counts, indicating that seasonality might be related to an increase in certain species of the genus. This investigation revealed that species from the *Fumigati* section were isolated in greater numbers during periods of drought, particularly in the winter. This trend is consistent with the characteristics of *A. fumigatus*, which thrives under such conditions [46]. *A. fumigatus* spores are highly conducive to air dispersion owing to their small size and significantly higher hydrophobicity than those of other *Aspergillus* species, allowing them to remain airborne for longer periods [6]. A study conducted in the USA reported that the incidence of aspergillosis was associated with seasonal periods of low precipitation and high temperatures. According to the authors, hot and dry weather conditions facilitate greater dispersal of hydrophobic conidia, which are critical factors in the transmission of aspergillosis [44].

In this study, *Aspergillus* isolates were initially identified based on their macro- and microscopic characteristics, and almost all were classified into sections. However, phenotypic identification of *Aspergillus* fungi is challenging and has certain limitations. This is because these fungi share many species similarities and can exhibit morphological variations dependent on culture medium or incubation temperature. Moreover, phenotypic identification requires analysts to have a certain level of experience [16].

**Figure 3:**
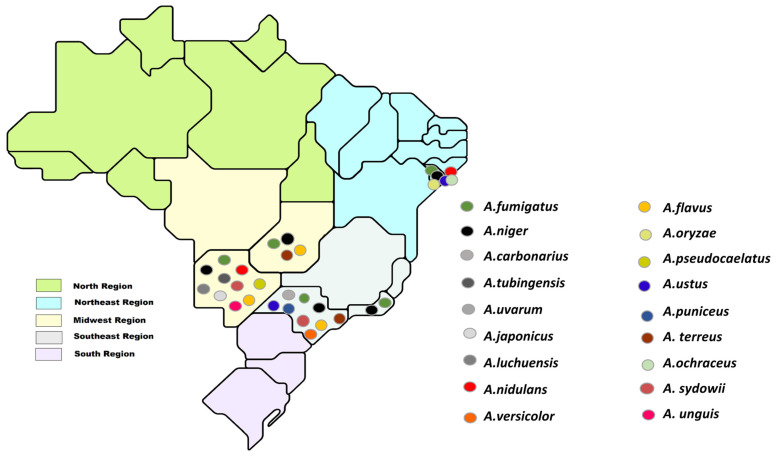
Distribution of species of *Aspergillus* identified in indoor air of Brazilian hospitals.

The results of this study help fill a gap in the knowledge of *Aspergillus* species present in the internal air of hospitals in Brazil. Figure 3 shows the distribution of *Aspergillus* species identified in the indoor air of hospitals in only three of the five regions of Brazil: Northeast [27,36], Midwest [37], and Southeast [38,47]. Despite their high sensitivity and specificity, molecular techniques are of moderate complexity, require specialized technical knowledge, and have high capital costs [48], which makes it challenging to implement the routine of hospital laboratories from low income regions.

Molecular identification revealed cryptic species (which are difficult to distinguish by morphological identification and exhibit distinctive molecular characteristics), including *A. sydowii* (n = 9), *A. tubingensis* (n = 4), *A. pseudotamarii* (n = 2), *A. luchuensis* (n = 1), *A. uvarum* (n = 1), *A. japonicus* (n = 1), and *A. pseudocaelatus* (n = 1) in the hospital’s indoor air. *A. sydowii* (*Nidulantes* section) and *A. tubingensis* (*Nigri* section) are among the most common *Aspergillus* cryptic species in human pathology [3,49,50]. *A. sydowii* is known to cause allergies and aggravate asthma, and is rarely involved in endophthalmitis, aspergilloma, and invasive pulmonary aspergillosis [34,50]. *A. tubingensis* is commonly found in indoor air and has been described as an agent of otitis, keratitis, and lower respiratory tract infections in hospitalized patients [3,7]. Nosocomial infections caused by these agents are difficult to treat because of their reduced sensitivity to amphotericin B and intrinsic resistance to azoles [3,10,49,50]. *A. luchensis*, another important member of the *Aspergillus* species belonging to the *Nigri* section, can cause invasive aspergillosis [51]. *A. pseudotamarii*, an aflatoxigenic member of the *Aspergillus* section *Flavi*, has been identified as a mycotic keratitis agent [52]. *A. japonicus* is one of the main allergenic fungi in India and is considered an agent of pulmonary aspergillosis in patients with COVID-19 [3,53,54]. *A. pseudocaelatus* is a toxigenic fungus that produces aflatoxins in various crops [55]. Improvements in microbiology laboratories and the widespread use of molecular diagnostic tools will facilitate more precise species descriptions [56].

## 5. Conclusions

*Aspergillus* fungi, including species from the *Fumigati*, *Nidulantes*, *Nigri*, and *Flavi* sections, were found in the indoor air of a tertiary referral hospital for infectious diseases in the mid-western region of Brazil.

Isolates from the *Fumigati* section were more prevalent during the winter months; however, additional research is needed to establish the seasonality of these fungi.

The presence of *A. fumigatus* in intensive care units increases the risk of aspergillosis caused by these pathogens, and emphasizes the need for measures to improve air quality in the hospital environment.

## Figures and Tables

**Table 1:** Primer used in DNA amplification and sequencing for molecular identification of *Aspergillus* species.

Primer	Sequence (5′-3′)	Reference
*β-tubulin 2a*	GGTAACCAAATCGGTGCTGCTTTC	Glass and Donaldson, 1995 [19]
*β-tubulin 2b*	ACCCTCAGTGTAGTGACCCTTGGC	Glass and Donaldson, 1995 [19]
*Calmodulin 1*	GARTWCAAGGAGGCCTTCTC	O’Donnell et al., 2000 [20]
*Calmodulin 2a*	TTTTTGCATCATGAGTTGGAC	O’Donnell et al., 2000 [20]
*Calmodulin 11*	ACCATGATGGCGCGCAAG	O’Donnell et al., 2000 [20]
*Calmodulin 22*	TCCTTCATCTTGCGCGCC	O’Donnell et al., 2000 [20]

**Table 2:** Comparison of filamentous fungal load according to collection date. UHMAP—Campo Grande, 2021.

	CFU/m^3^ of Filamentous Fungi/100 L/min		
Date	N (%)	Mean per Plate (SD)	*p* Value *
12 January	253 (6.04)	9.3 (6.6)	
26 January	448 (10.46)	16.7 (9.2)	
9 February	202 (4.98)	7.4 (5.0)	
23 February	325 (7.55)	12.5 (9.2)	
9 March	431 (10.33)	15.9 (7.8)	<0.001
6 August	442 (10.80)	17.0 (6.7)	
20 August	498 (12.22)	19.1 (8.9)	
7 December	593 (11.86)	18.8 (7.6)	
14 December	1103 (25.73)	42.4 (12.5)	

Note: SD = standard deviation; * ANOVA. CFU = Colony Forming Unit.

**Table 3:** Distribution of 324 *Aspergillus* species grouped by section, per day of collection. UHMAP—Campo Grande-MS, 2021.

Date	*Flavi* N (%)	*Nidulantes* N (%)	*Nigri* N (%)	*Terrei* N (%)	*Fumigati* N (%)	Indeterminate N (%)	Total of *Aspergillus* N (%)	*p* Value
12 January	0 (0.0)	0 (0.0)	5 (55.5)	0 (0.0)	4 (44.4)	0 (0.0)	9 (2.8)	<0.001
26 January	8 (17.8)	5 (11.1)	26 (57.8)	4 (8.9)	2 (4.4)	0 (0.0)	45 (13.9)	
9 February	0 (0.0)	26 (86.7)	4 (13.3)	0 (0.0)	0 (0.0)	0 (0.0)	30 (9.3)	
23 February	1 (2.6)	18 (47.4)	18 (47.4)	1 (2.6)	0 (0.0)	0 (0.0)	38 (11.7)	
9 March	11 (33.3)	0 (0.0)	18 (54.5)	2 (6.1)	0 (0.0)	2 (6.1)	33 (10.2)	
6 August	1 (1.5)	13 (27.6)	4 (6.0)	1 (1.5)	48 (71.6)	0 (0.0)	67 (20.7)	
20 August	9 (26.4)	3 (8.8)	5 (14.7)	1 (2.9)	16 (47.1)	0 (0.0)	34 (10.5)	
7 December	5 (11.9)	13 (30.9)	5 (11.9)	1 (2.4)	18 (42.9)	0 (0.0)	42 (13.0)	
14 December	3 (11.5)	12 (46.2)	4 (15.4)	0 (0.0)	7 (26.9)	0 (0.0)	26 (8.0)	
Total	38 (11.7)	90 (27.8)	89 (27.4)	10 (3.1)	95 (29.3)	2 (0.6)	324 (100)	

**Table 4:** *Aspergillus* sections scattered in the indoor air during the UHMAP season (Campo Grande-MS, 2021).

*Aspergillus* spp. (Section)	WINTER N (%)	SPRING N (%)	SUMMER N (%)	Total of *Aspergillus* N (%)	*p* Value
*Flavi*	10 (26.3)	8 (21.0)	20 (52.6)	38 (11.7)	<0.001
*Nidulantes*	16 (17.8)	25 (27.8)	49 (54.4)	90 (27.8)	
*Nigri*	9 (10.1)	9 (10.1)	71 (79.8)	89 (27.4)	
*Terrei*	2 (20.0)	1 (10.0)	7 (70.0)	10 (3.1)	
*Fumigati*	64 (67.4)	25 (26.3)	6 (6.3)	95 (29.3)	
Indeterminate	0 (0.0)	0 (0.0)	2 (100.0)	2 (0.6)	
Total	101 (31.2)	68 (21.0)	155 (47.8)	324 (100)	

## Data Availability

Data are contained within the article.
